# Sulfate-Reducing Bacteria Isolated from an Oil Field in Kazakhstan and a Description of *Pseudodesulfovibrio karagichevae* sp. nov.

**DOI:** 10.3390/microorganisms12122552

**Published:** 2024-12-11

**Authors:** Salimat K. Bidzhieva, Tatyana P. Tourova, Denis S. Grouzdev, Salima R. Samigullina, Diyana S. Sokolova, Andrey B. Poltaraus, Alexander N. Avtukh, Vera M. Tereshina, Andrey V. Mardanov, Nurlan S. Zhaparov, Tamara N. Nazina

**Affiliations:** 1 Winogradsky Institute of Microbiology, Research Center of Biotechnology, Russian Academy of Sciences, Moscow 119071, Russia; salima.bidjieva@gmail.com (S.K.B.); tptour@rambler.ru (T.P.T.); samigullinasalimar@gmail.com (S.R.S.); sokolovadiyana@gmail.com (D.S.S.); v.m.tereshina@inbox.ru (V.M.T.); 2 SciBear OU, Tartu mnt 67/1-13b, 10115 Tallinn, Estonia; denisgrouzdev@gmail.com; 3 Engelhardt Institute of Molecular Biology, Russian Academy of Sciences, Moscow 119991, Russia; abpolt@gmail.com; 4 Skryabin Institute of Biochemistry and Physiology of Microorganisms, Russian Academy of Sciences, Pushchino Scientific Center for Biological Research of the Russian Academy of Sciences, Pushchino, Moscow 142290, Russia; avtukh@rambler.ru; 5 Institute of Bioengineering, Research Center of Biotechnology, Russian Academy of Sciences, Moscow 119071, Russia; mardanov@biengi.ac.ru; 6 Branch of the Limited Liability Partnership “KazMunaiGas Engineering”, Aktau 130000, Kazakhstan; zhaparov_n@kaznipi.kz

**Keywords:** *Pseudodesulfovibrio*, sulfate-reducing bacteria, polyphasic taxonomy, functional genomics, unique genes, petroleum reservoir

## Abstract

Sulfidogenic bacteria cause numerous issues in the oil industry since they produce sulfide, corroding steel equipment, reducing oil quality, and worsening the environmental conditions in oil fields. The purpose of this work was to isolate and taxonomically identify the sulfidogenic bacteria responsible for the corrosion of steel equipment at the Karazhanbas oil field (Kazakhstan). In this study, we characterized five sulfidogenic strains of the genera *Pseudodesulfovibrio*, *Oleidesulfovibrio*, and *Acetobacterium* isolated from the formation water of the Karazhanbas oil field (Kazakhstan). Sulfate-reducing strain 9FUS^T^ revealed 98.9% similarity of the 16S rRNA gene sequence with the closely related strain ‘*Pseudodesulfovibrio methanolicus*’ 5S69^T^ and was studied in detail to enhance the taxonomic resolution. Strain 9FUS^T^ grew optimally at 23–28 °C, pH 6.5, and 0–2% (*w*/*v*) NaCl. The strain used lactate, pyruvate, methanol, ethanol, fructose, ribose, and H_2_/CO_2_ (in the presence of acetate) as carbon and energy sources for sulfate reduction. *Iso*-C_17:1_ ω11, C_15:0_, *iso*-C_15:0_, and C_16:0_ were the predominant fatty acids. The genome is 4.20 Mbp with a G + C content of 64.0%. The average nucleotide identity and digital DNA–DNA hybridization values with *Pseudodesulfovibrio* spp. genomes were 72.5–91.6% (<95%) and 18.5–45.0% (<70%), respectively, and supported our conclusion that 9FUS^T^ (=VKM B-3654^T^ = KCTC 25498^T^) belonged to a novel *Pseudodesulfovibrio* species, for which the name *Pseudodesulfovibrio karagichevae* sp. nov. is proposed. Pangenome analysis of sixteen *Pseudodesulfovibrio* species and functional annotation analysis of identified genes revealed complete modules of enzymes of the main metabolic pathways, characteristic of bacteria of this genus, and unique genes highlighting the adaptations of strain 9FUS^T^ in carbohydrate metabolism, nutrient uptake, and environmental stress response. Isolation of these strains expands our understanding of the diversity of sulfidogens in oil reservoirs and can be used to test the effectiveness of biocides used in an oil field.

## 1. Introduction

Corrosion of steel equipment is an important problem in the water supply system, as well as in the oil and gas industry [1,2]. For example, in 2015, global losses due to corrosion amounted to about 2.5 trillion dollars [3]. The main agents of microbially (microbiologically) influenced corrosion (MIC) of steel equipment are sulfate-reducing prokaryotes (SRP): bacteria (SRB) and archaea (SRA), which reduce sulfate of the reservoir water to form sulfide [4,5,6]. In petroleum reservoirs, the substrates for SRP are organic acids and alcohols dissolved in reservoir water, petroleum hydrocarbons, as well as molecular hydrogen/CO_2_ [7,8,9]. In the absence of sulfates, SRPs are able to ferment organic substrates or grow syntrophically using methanogens as biological electron acceptors. Hydrogen sulfide is a toxic gas; it dissolves in reservoir water and oil and causes environmental problems during oil extraction, transportation, and refining. The mechanisms of microbially influenced corrosion are discussed in the review [6]. High rates of sulfate reduction in oil fields with sulfate-containing formation water, as well as in those flooded with seawater, were registered by radiotracer techniques [10,11]. Along with SRP, corrosion is also caused by microorganisms that reduce other oxidized sulfur compounds (thiosulfate-, sulfite-, and sulfur-reducing), iron-reducing, iron-oxidizing, and organic acid-producing fermentative bacteria and methanogenic archaea [12,13,14]. Hydrogenotrophic homoacetogens and methanogens consume cathodic hydrogen from Fe with the formation of acetate and methane, respectively, and contribute to the corrosion process.

This study is part of the work aimed at identifying the potential producers of hydrogen sulfide and corrosion agents at the Karazhanbas oil field (Kazakhstan) using molecular and microbiological approaches [15]. The oil-bearing horizons of the Karazhanbas field are located at shallow depths (250–500 m) and vary in temperature from 25 to 45 °C. A feature of the Karazhanbas field is the presence of high-viscosity sulfurous oil. Although sulfate was not registered in formation and injection water at the time of sampling, earlier it was detected at low concentrations (10–69 mg·L^−1^) in formation water, and corrosion of oil-processing equipment and sulfide release into the produced fluids were observed at the oilfield [16].

In a previous study [15], using a molecular approach based on metabarcoding of V3–V4 regions of the 16S rRNA genes, methanogenic archaea of the genera *Methanococcus*, *Methanobacterium*, and *Methanothrix*; syntrophic bacteria of the genus *Smithella*; as well as bacteria of the genera *Arcobacter*, *Acetobacterium*, *Marinobacter*, *Thiomicrospira*, and *Thermovirga* were detected in formation water. The share of sulfate-reducing bacteria in water samples was low (<1% total). Mesophilic bacteria of the genera *Pseudodesulfovibrio*, *Desulfovibrio*, *Desulfosarcina*, *Desulfoglaeba*, and *Desulfotignum*, and thermophilic bacteria of the genera *Thermodesulfobacterium*, *Thermodesulfovibrio*, and *Defluviitoga* were detected by the molecular approach in formation water and sulfidogenic enrichments. Prokaryotes revealed in formation water samples by this approach were potentially able to carry out the key processes (sulfate reduction, methane formation, and degradation of hydrocarbons).

The purpose of this work was the isolation and identification of pure cultures of sulfidogenic bacteria from the formation water of the Karazhanbas oil field (Kazakhstan) and the characterization of new isolates, as well as sequencing and analysis of the strain 9FUS^T^ genome to clarify its potential functional role in the subsurface environment. In this study, using different electron donors and acceptors, five strains of sulfate-reducing bacteria and an acetogenic strain were isolated and identified. The results of phenotypic studies, analysis of the genome, and unique genes allowed us to assign the sulfate-reducing strain 9FUS^T^ to the genus *Pseudodesulfovibrio* and to provide a description of a new species, *Pseudodesulfovibrio karagichevae* sp. nov. The isolated strains can be used to select effective biocides for injection into the oil reservoir

## 2. Materials and Methods

### 2.1. Source of Enrichment and Pure Cultures

Sulfidogenic bacteria were isolated from formation water collected in June 2019 at the Karazhanbas oil field (Buzachi Peninsula, Mangystau Province, Kazakhstan). The water sample was obtained from the production well 6069 (43°22′33.3″ N, 52°59′27.1″ E), exploiting the Lower Cretaceous layer G, located at 350 m below sea level and having a temperature of 25 °C. Formation water belonged to the calcium-chloride type and had a total salinity (of 32,959.2 mg·L^−1^) and pH 6.6; while sulfate was not registered in this water sample at the moment of investigation, sulfide was detected at a concentration of 62.9 mg·L^−1^ (Table S1). The G horizon is composed mainly of loose sands and weakly cemented sandstones with interlayers of red-brown clays; reservoir porosity is 35%, and permeability is 125 mD. The oil of this deposit has a density of 939–944 kg·m^−3^ and high viscosity (160–660 mPa·s at 50 °C). It contains high levels of sulfur (1.6–2.2 mass %), resins (24 mass %), asphaltenes (24.9–29.1 mass %), and paraffin (0.7–1.4 mass %). There is a significant content of trace elements in oil, in particular, vanadium pentoxide −308.6 g·t^−1^. The average gas content is 8.9–9.8 m^3^ per 1 t oil [16]. Well, 6069 is located in an area flooded with production water re-injected into the reservoir (PWRI) after oil separation. Injection water contained both sulfate (14 mg·L^−1^) and sulfide (25 mg·L^−1^). The physicochemical and microbiological characteristics of the oil field were described previously [15].

### 2.2. Isolation, Media Composition and Phenotypic Characterization

Five strains of sulfate-reducing bacteria (9FUS^T^ (=VKM B-3654^T^ = KCTC 25,498^T^), 9ES, 09, DNS2, 09S) and one strain of sulfur-reducing bacteria (9FOS) were isolated from the Karazhanbas oil field. For enrichment, isolation, and cultivation of sulfate-reducing bacteria, the following basal mineral medium was used (per liter distilled water): 0.2 g KH_2_PO_4_, 0.25 g NH_4_Cl, 2.0 g NaCl, 0.4 g MgCl_2_·6H_2_O, 0.5 g KCl, 0.1 g CaCl_2_·2H_2_O, and 0.5 g Na_2_S·9H_2_O. The medium was amended with 0.2 g of yeast extract, 1 mL of 0.1% (*w*/*v*) Mohr’s salt solution (FeSO_4_·(NH_4_)_2_SO_4_·6H_2_O), and 1 mL each of vitamin [17] and microelements solutions [18]. The medium was reduced with cysteine-HCl; the pH of the medium was adjusted to pH 6.5 with the addition of HCl (10%) or NaOH (10%) at 25 °C.

The medium was prepared anaerobically under a stream of Ar, dispensed into Hungate tubes [19], sealed with butyl rubber stoppers and plastic caps, and autoclaved at 121 °C for 60 min. Sulfate-reducing enrichments were obtained in a basal medium supplemented with 3.5 g sodium lactate and 2 g sodium sulfate per liter. Pure cultures were isolated by the method of successive transfers of grown cultures from the highest growth-positive dilutions (10^−7^–10^−8^) on a fresh medium of the same composition. The cultures were incubated at a temperature of 23–25 °C. Replacement of lactate with other substrates [ethanol (2 mL·L^−1^), formate (2 g·L^−1^), fumarate (2 g·L^−1^), H_2_/CO_2_ (4:1, *v*/*v*)] and sulfate with elemental sulfur (2.5 g·L^−1^) allowed us to isolate a number of other sulfidogenic bacteria. The purity of the cultures was checked by phase-contrast microscopy of wet biomass and by 16S rRNA sequencing of the total liquid culture from the tube. Growth was monitored by optical density (OD_600_) measurements and by sulfide production, which was determined using the colorimetric method [20].

Strain 9FUS^T^ was studied simultaneously with the type strain 5S69^T^ of the new species ‘*Pseudodesulfovibrio methanolicus*’, which was used for comparison [21]. Cell morphology, physiology, DNA isolation, 16S rRNA gene sequencing, composition of cellular fatty acids, phospholipids, and isoprenoid quinones were analyzed as described previously [21].

### 2.3. Genome Sequencing and Annotation

The genomic DNA of strain 9FUS^T^ was isolated using the DNeasy PowerSoils Kit (Qiagen, Düsseldorf, Germany). The library for Illumina sequencing was constructed using the NEBNext Ultra II DNA Library Prep Kit (New England Biolabs, Ipswich, MA, USA). Illumina MiSeq run output was 3,031,658 paired-end reads (2 × 300 nt, ~874 Mbp in total). FLASH v.1.2.11 [22] was used to merge paired-end reads into longer sequences, and low-quality read fragments were removed with Sickle v.1.33 (https://github.com/najoshi/sickle, accessed on 28 January 2023) [23]. The processed reads were assembled into contigs by SPAdes v. 3.15.4 [24] in isolate mode. The NCBI Prokaryotic Genome Annotation Pipeline (PGAP), executed as a part of the submission process to the NCBI GenBank database, produced genome annotation. All software was run using default settings.

### 2.4. Genomic and Phylogenetic Analyses

Pairwise average nucleotide identity (ANI) and digital DNA-DNA hybridization (dDDH) values were calculated using FastANI v1.3 [25] and the Genome-to-Genome Distance Calculator v3.0 [26], respectively. Phylogenomic analysis of *Pseudodesulfovibrio* genomes utilized a concatenated alignment of 120 single-copy phylogenetic marker genes obtained via GTDB-Tk v1.4.0 [27]. A maximum-likelihood (MLH) tree of the 16S rRNA gene sequences was constructed using a GTR + F + I + G4 model, while the phylogenomic tree was built using an LG + F + I + G4 model as recommended by ModelFinder [28] in IQ-TREE [29]. Branching supports were evaluated with 10,000 ultrafast bootstraps [30]. Maximum parsimony (MP) and neighbor-joining (NJ) trees were reconstructed using MPBoot [31] and MEGA11 [32], respectively. Pangenomic analysis was conducted using the bioinformatics pipeline suggested previously [33] and Anvi’o v8.0 [34]. Genomes were organized based on the distribution of gene clusters using the MCL algorithm (Euclidean distance, Ward linkage). Orthologous Groups (COG) of proteins were predicted and classified using COGclassifier v1.0.5 (https://github.com/moshi4/COGclassifier, accessed on 7 September 2022). Potential metabolic pathways were reconstructed by comparing *Pseudodesulfovibrio* genomes using the BlastKOALA tool [35] of KEGG v3.0 [36].

### 2.5. Nucleotide Sequence Accession Numbers

The GenBank accession numbers for the 16S rRNA gene sequences of six strains are PQ453521–PQ453525 and PQ474646. The genome sequence of strain 9FUS^T^ has been deposited at GenBank under the accession number GCF_041414475.1.

## 3. Results and Discussion

This article describes six strains of anaerobic sulfidogenic bacteria isolated from the Karazhanbas oil field (Kazakhstan). The strains were identified by 16S rRNA gene analysis. To clarify the taxonomic position and the potential functional role in the underground habitat, the morphology, physiology, and chemotaxonomic features of the strain 9FUS^T^ were studied in detail, and its genome was sequenced and annotated, which made it possible to describe the new species *Pseudodesulfovibrio karagichevae* sp. nov.

### 3.1. Isolation and Identification of Pure Cultures from the Oil Field

Using media with different electron donors and acceptors, six pure cultures of mesophilic obligately anaerobic sulfidogenic bacteria were isolated from reservoir water samples taken at the Karazhanbas oil field. To isolate the strains, the mineral medium base was used, to which formate, acetate, ethanol, fumarate, or H_2_/CO_2_ were added as electron and carbon donors; sulfate, elemental sulfur, and fumarate served as electron acceptors.

The isolated strains (9FUS^T^, 9ES, 09, DNS2, 09S, and 9FOS) reduced sulfate and/or elemental sulfur with sulfide formation. In addition to the substrates listed in Table 1, all five sulfate-reducing strains (9FUS^T^, 9ES, 09, DNS2, and 09S) grew on a medium with H_2_/CO_2_ supplemented with acetate, forming sulfide, and strain 9FOS was a homoacetogen, growing on a mixture of H_2_/CO_2_ in the absence of an electron acceptor, forming acetic acid.

A high level of the 16S rRNA gene sequence similarity (99–100%) of four strains with the GeneBank sequences allowed the strain DNS2 to be classified as *Oleidesulfovibrio alaskensis* (previously *Desulfovibrio alaskensis* [37,38] and the strain 9FOS—to the species *Acetobacterium carbinolicum* [39,40]. The 16S rRNA gene sequences of strains 9FUS^T^, 9ES, 09S, and 09 had 100% similarity to each other and 98.4–98.7% similarity to the gene *Pseudodesulfovibrio mercurii* ND132^T^ [41].

These results indicated that strains 9FUS^T^, 9ES, and 09 could represent a novel species of the genus *Pseudodesulfovibrio*. The phenotypic features of the strain 9FUS^T^, selected as the type strain, were investigated, and its genome was sequenced and analyzed.

### 3.2. Physiological and Chemotaxonomic Characterization of the Strain 9FUS^T^

**Figure 1:**
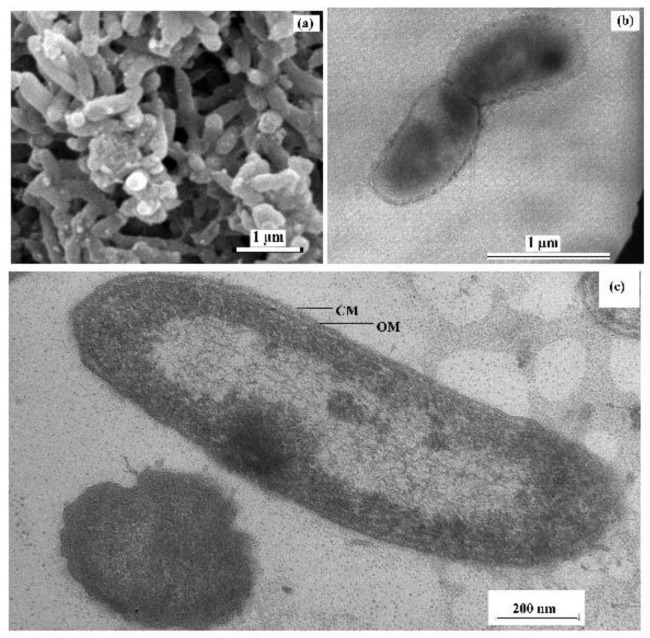
Scanning electron micrograph (**a**) and transmission electron micrographs of negatively stained cells with a flagellum (**b**) and an ultrathin section of a cell of strain 9FUS^T^ showing the visible cellular membrane (CM) and outer membrane (OM) divided by the periplasmic space (**c**). The strain was grown in the lactate/sulfate medium for 7 days at 25 °C.

Cells of the sulfate-reducing strain 9FUS^T^ were non-spore-forming, curved rods, 0.3–0.5 µm in diameter and 1–2 µm long, motile in the exponential growth phase due to a single polar flagellum (Figure 1a,b). The cells were Gram-stain-negative and had Gram-negative cell wall structure, which was confirmed by the presence of an inner cytoplasmic membrane and an outer lipoprotein membrane on microphotographs of ultrathin cell sections (Figure 1c).

In the lactate/sulfate medium, strain 9FUS^T^ grew at the temperature range from 15 to 37 °C with an optimum at 23–28 °C (Figure S1a). No growth was observed at 10 and 42 °C. NaCl range was 0–5% (*w*/*v*) with a growth optimum at 0–2% (*w*/*v*) NaCl (Figure S1b). The pH range for growth was 4.1–8.6, with optimum pH around 6.5 (Figure S1c). However, the pH of the medium, which was 4.1–5.6 at the beginning of incubation, increased by the end of incubation to pH 6.5 (Table 2).

In sulfate-containing media with yeast extract (0.2 g·L^−1^), strain 9FUS^T^ used lactate, pyruvate, formate, fumarate, succinate, malate, methanol, ethanol, glycerol, and fructose; weak growth was observed on tryptone, ribose, and galactose, while the strain did not use benzoate, acetate, propionate, butyrate, lactose, glucose, mannose, glutamate, glycine, methylamine hydrochloride, or citrate. The strain was not capable of autotrophic growth on H_2_/CO_2_ but grew on H_2_/CO_2_ in the presence of acetate as a carbon source for constructive needs. The strain fermented pyruvate in the absence of sulfate (cultivation time 15 days at 25 °C) to form ethanol (1 mM), acetate (12 mM), CO_2_ (6% of the volume of the gas phase), and H_2_ (11%, *v*/*v*). Sulfate, sulfite, thiosulfate, elemental sulfur, and fumarate, but not nitrate, were used as terminal electron acceptors in the presence of lactate.

The predominant fatty acids in strain 9FUS^T^ were *iso*-C_17:1_ ω11 (20.8%), C_15:0_ (14.6%), *iso*-C_15:0_ (9.0%), C_16:0_ (8.5%), and C_17:0_ (7.7%) (Table S2). This fatty acid profile was different from that of ‘*Pseudodesulfovibrio methanolicus*’ 5S69^T^. The major polar lipids in strain 9FUS^T^ were phosphatidylethanolamine, diphosphatidylglycerol, and phosphatidylglycerol; among the minor lipids, unidentified glycolipid and aminophosholipids were detected (Figures S2 and S3). The main respiratory quinone of strain 9FUS^T^ was MK-6(H_2_). Physiological and biochemical characteristics that distinguish strain 9FUS^T^ from phylogenetically closely related species of the genus *Pseudodesulfovibrio* are summarized in Table 2.

### 3.3. Phylogenetic Analysis of 16S rRNA Gene Sequences

**Figure 2:**
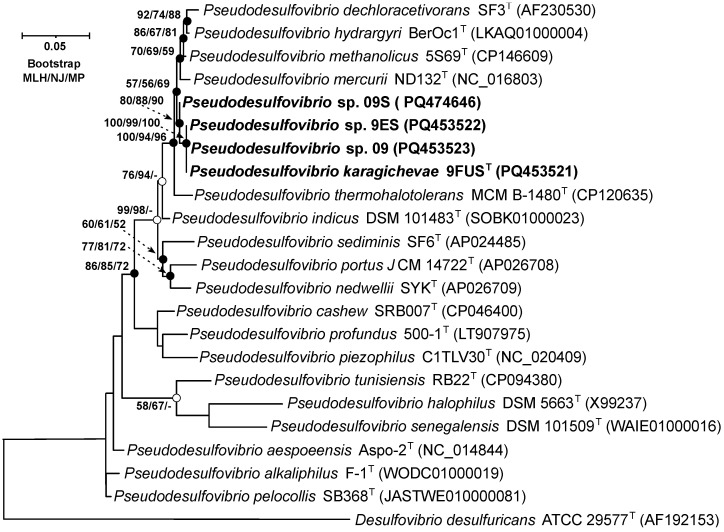
Maximum-likelihood phylogenetic tree based on 16S rRNA gene sequences (1444 nucleotide sites), demonstrating the position of strain 9FUS^T^ within the genus *Pseudodesulfovibrio*. White circles indicate that the relevant nodes were recovered using the neighbor-joining algorithm; black circles indicate that the relevant nodes were also recovered based on the neighbor-joining and maximum-parsimony algorithms. Bootstrap values (>50%) are listed as percentages at the branching points. Bar, 0.05 substitutions per nucleotide position. The tree was rooted using *Desulfovibrio desulfuricans* ATCC 29577^T^ as the outgroup. GenBank accession numbers for 16S rRNA genes are indicated in parentheses. The name of the studied strain is marked in boldface.

On the 16S rRNA gene phylogenetic tree, constructed using the MLH, NJ, and MP methods (Figure 2), strain 9FUS^T^ clustered within a well-supported clade, which included ‘*P. methanolicus*’ 5S69^T^, *P. hydrargyri* BerOc1^T^, *P. mercurii* ND132^T^, ‘*P. thermohalotolerans*’ MCM B-1480^T^, and *P. indicus* J2^T^. These results confirm that strain 9FUS^T^ belongs to the genus *Pseudodesulfovibrio*. Comparison of the 16S rRNA gene sequences of strain 9FUS^T^ revealed that the strain had similarities ranging from 92.8% to 98.9% with members of the genus *Pseudodesulfovibrio* (Table 3). However, it was impossible to definitively determine whether strain 9FUS^T^ represents a new species, as its 16S rRNA gene sequence is similar to ‘*P. methanolicus*’ 5S69^T^, which exceeds 98.7% [44,45], which is the typical threshold for species delineation. To resolve this, whole-genome sequencing and further comparative genomic analyses were performed to assess additional genomic features and clarify the taxonomic position of strain 9FUS^T^.

### 3.4. Genome Features and Phylogeny

The genome of strain 9FUS^T^ was composed of 142 contigs with a total genomic length of 4,201,585 bp, an N50 value of 50.2 kb, a G + C content of 64.0%, and coverage of 132.0×. The genome comprises 3944 annotated genes, including 3851 protein-coding sequences, 23 pseudogenes, and 64 RNA genes. The genome of strain 9FUS^T^ shows ANI values ranging from 72.5% to 91.6% and dDDH values from 18.5% to 45.0% when compared with the genomes of the type strains of the genus *Pseudodesulfovibrio* (Table 3). These values are below the established thresholds of 95–96% for ANI and 70% for dDDH, which are generally accepted for species delineation [44,45]. This indicates that strain 9FUS^T^ represents a novel species within the genus *Pseudodesulfovibrio*.

**Figure 3:**
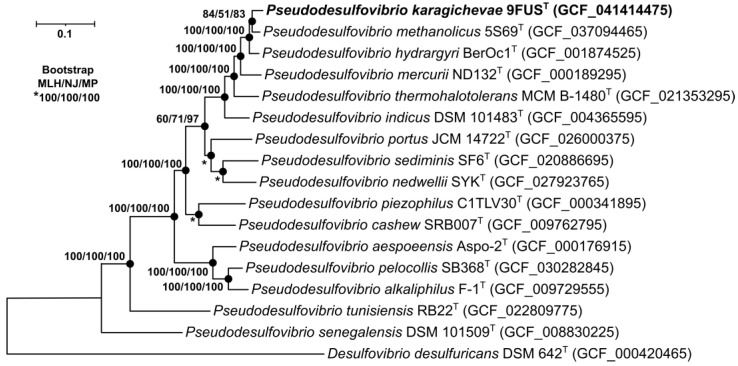
The maximum-likelihood phylogenetic tree derived from concatenated 120 single-copy proteins demonstrates the position of strain 9FUS^T^ within the genus *Pseudodesulfovibrio.* For phylogenetic analysis, 37,730 amino acid positions were used. Black circles indicate that the corresponding nodes were also recovered based on the neighbor-joining and maximum-parsimony algorithms. Bar, 0.05 amino acid substitutions per site. Bootstrap values (>50%) are listed as percentages at the branching points. The tree was rooted using *Desulfovibrio desulfuricans* DSM 624^T^ as the outgroup. Accession numbers for the genomic assemblies are indicated in parentheses. The name of the studied strain is marked in boldface.

To identify the phylogenetic position of strain 9FUS^T^, a maximum-likelihood tree was constructed based on the concatenated alignment of 120 single-copy proteins comprising 37,730 amino acid positions (Figure 3). On the phylogenetic tree, strain 9FUS^T^ formed a separate branch with ‘*P. methanolicus*’ 5S69^T^, *P. hydrargyri* BerOc1^T^, *P. mercurii* ND132^T^, and ‘*P. thermohalotolerans*’ MCM B-1480^T^.

**Figure 4:**
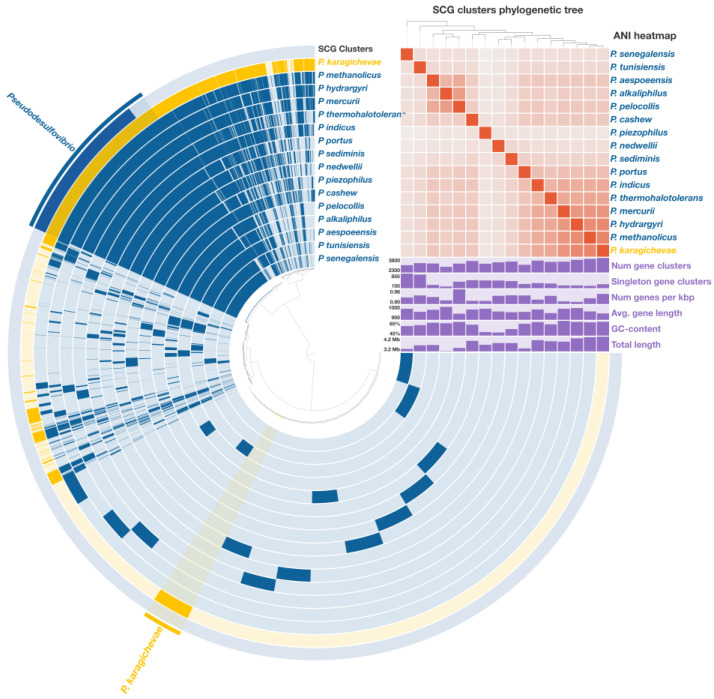
Pangenome analysis of sixteen *Pseudodesulfovibrio* species calculated with Anvi’o v. 8.0. The central dendrogram illustrates the relationships between 12,348 gene clusters (55,478 genes) across the analyzed genomes. Dark circular regions indicate the presence of specific genes in each genome. A phylogenetic tree was reconstructed based on single-copy genes, providing insights into the evolutionary relationships among the species. ANI heatmap illustrates similarity values ranging from 70% to 100%.

A comprehensive pangenomic analysis was performed on sixteen *Pseudodesulfovibrio* species, encompassing a total of 55,478 genes, which were grouped into 12,348 gene clusters (GCs). Among these, 1472 core gene clusters were consistently found across all analyzed genomes, including 1311 single-copy genes (SCGs) (Figure 4). The phylogenetic tree, reconstructed from these SCGs and covering 429,648 amino acid positions, exhibited a topology highly consistent with the phylogenomic tree derived from the concatenated analysis of 120 single-copy proteins. This robust congruence further supports the phylogenetic placement of strain 9FUS^T^ within the genus *Pseudodesulfovibrio*.

### 3.5. Genome Insights and Unique Genes of Strain 9FUS^T^

**Figure 5:**
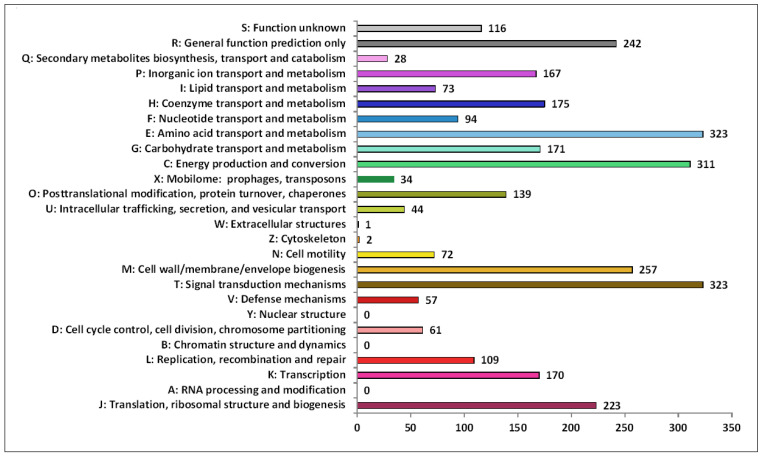
Functional classification in Clusters of Orthologous Groups of proteins (COG). The *X*-axis indicates the number of genes in functional categories.

The functional annotation analysis of identified genes in the 9FUS^T^ genome using the COG database revealed a total of 82.89% (3192 of 3851) sequences classified into the COG functional categories and assigned to 23 functional groups (Figure 5). Among the groups, signal transduction mechanisms (10.1%), which allow cells to translate signals from the extracellular environment into changes within the cell, amino acid transport and metabolism (10.1%), and energy production and conversion (9.7%) were the most abundant.

According to KEGG analysis of the genome of strain 9FUS^T^, its genome contains the genes encoding complete modules of enzymes of the main metabolic pathways, characteristic of bacteria of the genus *Pseudodesulfovibrio.* Using the BlastKOALA tool of KEGG, the main metabolic pathways of strain 9FUS^T^ were shown to coincide with those described earlier for the closely related ‘*P. methanolicus*’ 5S69^T^ [21]. Physiological and genomic characteristics of strain 9FUS^T^ were very similar to those of the type strain ‘*Pseudodesulfovibrio methanolicus*’ 5S69^T^, for which a detailed genomic analysis has recently been performed [21]. Both strains, as well as other bacteria of the genus *Pseudodesulfovibrio*, contained genes responsible for dissimilatory reduction in sulfate to sulfide; biosynthesis of ATP, amino acids, lipopolysaccharides, terpenoids, and menaquinones; metabolism of purines, pyrimidines, fatty acids, and hydrogen utilization. The genes coding complete modules of central carbohydrate metabolism, including the Embden–Meyerhof pathway, the Pentose phosphate pathway, the Leloir pathway of galactose degradation, and others, were revealed. In order not to duplicate the description of genes common to the genus and both new strains, 5S69^T^ and 9FUS^T^, an analysis of unique genes inherent only to the studied strain 9FUS^T^ was performed.

In this analysis, individual enzymes were identified that were specific to the metabolism of the studied strain and distinguished it from other members of the genus *Pseudodesulfovibrio* and, first of all, from the strain ‘*P. methanolicus*’ 5S69^T^.

The genome of strain 9FUS^T^ possessed 340 GCs unique to other *Pseudodesulfovibrio* species, 43 of which had a predicted function according to the KEGG database. This unique gene set highlights the specialized adaptations of strain 9FUS^T^, particularly in carbohydrate metabolism, nutrient uptake, and environmental stress response. Among the distinct features, genes encoding the key enzymes for carbohydrate processing, such as *galU* (AB6M95_09405, 04270) coding UTP-glucose-1-phosphate uridylyltransferase (EC: 2.7.7.9), suggest enhanced capabilities for polysaccharide biosynthesis and utilization of diverse carbon sources and ability to survive in stressful environments [64].

Among the unique genes in the genome of strain 9FUS^T^, the *ccoN1* gene (encoding the main cytochrome oxidase subunit cbb3-type subunit I (EC: 7.1.1.9)) was annotated, although the auxiliary genes of the *ccoNOQP* operon, encoding the remaining cbb3 oxidase subunits, were absent in the genome. This enzyme catalyzes aerobic respiration, but it can also function under anaerobic conditions. This has been shown for *Pseudomonas aeruginosa* PAO1, which performs denitrification [65]. The *ccoN1* gene has also been annotated in other sulfate reducers. The product of the *ccoN1* gene of strain 9FUS^T^ has 70.1% similarity of amino acid sequences with a similar product of the *Paucidesulfovibrio longus* strain DSM 6739^T^, which was also isolated from an oil-producing well. However, what function this product can perform in the metabolism of these strains remains unclear.

The unique *hddA* (AB6M95_09420) and *hddC* (AB6M95_09405) genes coding for the biosynthesis of nucleotide-activated glycero-manno-heptose precursors of bacterial glycoproteins and cell surface polysaccharides were revealed [66].

The genome 9FUS^T^ also harbors unique genes involved in DNA repair and stress response, such as *dcm* (AB6M95_04415), encoding DNA (cytosine-5)-methyltransferase (EC: 2.1.1.37) [67], and *uvrD* (AB6M95_08355), encoding ATP-dependent DNA helicase (K03657) [68]. The strain has notable genomic elements related to horizontal gene transfer and genome maintenance, as evidenced by the presence of integrase/recombinase genes *xerC* (AB6M95_00800) and *xerD* (AB6M95_15490), which may contribute to genetic plasticity and adaptability [69].

Unlike the ‘*P. methanolicus*’ 5S69^T^, the strain 9FUS^T,^ was able to grow on ribose. Ribose is incorporated into the pentose phosphate cycle by the enzyme ribokinase (EC: 2.7.1.15), which catalyzes D-ribose phosphorylation to D-ribose 5-phosphate.

The ability to use ribose as a substrate of strain 9FUS^T^ is confirmed by the annotation of the *rbsK* gene (AB6M95_08240) encoding ribokinase in its genome (Figure S4).

**Figure 6:**
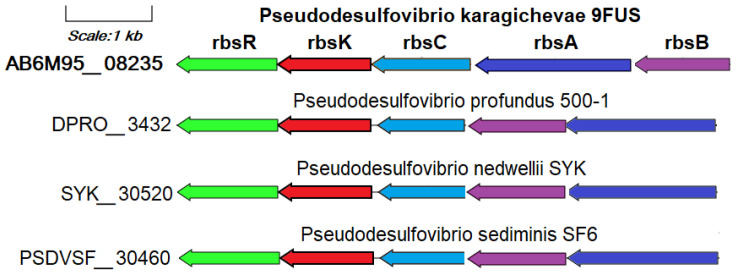
The genes presumably encoding ribose metabolism in the genome of the strain 9FUS^T^. Abbreviations: *rbsK*, ribokinase; *rbsA*, ribose transport system ATP-binding protein; *rbsB*, ribose transport system substrate-binding protein; *rbsC*, ribose transport system permease protein; *rbsR*, DNA-binding transcriptional repressor of ribose metabolism.

This gene is part of the ribose operon *rbsBACKR* (AB6M95_08235-08260), together with the unique genes identified in the pangenomic analysis of the ribose ATP-binding cassette transport system: *rbsA* (ribose transport system ATP-binding protein RbsA, EC: 7.5.2.7), *rbsB* (ribose transport system substrate-binding protein RbsA), and *rbsC* (ribose transport system permease protein RbsC), as well as the *rbsR* gene encoding DNA-binding transcriptional repressor of ribose metabolism (Figure 6). The ribose transport system proteins RbsA, RbsB, and RbsC work together to import ribose efficiently, particularly in nutrient-limited environments, by leveraging the energy from ATP hydrolysis for active transport across the membrane [70]. In addition to the genome of strain 9FUS^T^, the ribose metabolism operon is annotated only in the genomes of *P. profundus* 500^T^, *P. nedwellii* SYK^T^, and *P. sediminis* SF6^T^. The use of ribose as a substrate is a rare property for bacteria of the genus *Pseudodesulfovibrio* that is not related to the source of their allocation.

Strain 9FUS^T^, similar to *P. methanolicus* 5S69^T^, was able to grow on fructose as a sole carbon source. According to the BlastCOALA tool, this ability of strain 9FUS^T^ is presumably provided by the fructose catabolism gene *scrK* (V8V93_00875), encoding fructokinase (EC: 2.7.1.4), which catalyzes phosphorylation of D-fructose to D-fructose 6-phosphate. This enzyme has been studied mainly for eukaryotes, but it also functions in bacteria. In particular, for *Bifidobacterium longum* DSM 20219^T^, it was shown that the enzyme is necessary for the assimilation of fructose [71]. Subsequently, D-fructose 6-phosphate is incorporated into glycolysis via D-fructose 1,6-bisphosphate. However, in strain 9FUS^T,^ another fructose assimilation pathway presumably additionally functions, involving the enzyme 1-phosphofructokinase (EC: 2.7.1.56), the one most widely used by bacteria, animals, and plants (Figure S5). The action of this enzyme is provided by the fructose operon genes annotated in the genome of strain 9FUS^T^, including *fruK* (AB6M95_07015), encoding 1-phosphofructokinase (EC: 2.7.1.56); *fruA* (AB6M95_07020), encoding the fructose PTS system EIIBC or EIIC component (EC: 2.7.1.202); and *fruB* (AB6M95_07010), encoding a multiphosphoryl transfer protein (phosphoenolpyruvate–protein phosphotransferase; EC: 2.7.3.9). Among all members of the genus *Pseudodesulfovibrio*, duplicated genetic structures potentially capable of fructose assimilation, except for the genome of strain 9FUS^T^, have been annotated only in the genomes of *P. hydrargyri* BerOc1^T^ and *P. sediminis* SF6^T^.

The ability of strain 9FUS^T^ to grow on glycerol is confirmed by the annotation in its genome of a complete cluster of glycerol metabolism genes *glpK*-*hdrBC-glpBARSTPQUV* (AB6M95_08170-08225), homologous to the one previously described for *P. methanolicus* 5S69^T^, a strain also capable of glycerol utilization [21]. However, glycerol utilization by strain 9FUS^T^ may also probably follow another pathway due to the presence of the *adh* gene (AB6M95_01280) coding alcohol dehydrogenase (NADP+) (EC: 1.1.1.2); this gene has not been annotated in the genome of *P. methanolicus* 5S69^T^. This enzyme may catalyze the oxidation of alcohols, including glycerol, which is oxidized to D-glyceraldehyde; the latter is subsequently catabolized via the intermediate metabolites D-glycerate and 2-phospho-D-glycerate with involvement in glycolysis (Figure S6).

According to the BlastKOALA data, strain 9FUS^T^, similar to most *Pseudodesulfovibrio* species, is capable of dinitrogen fixation with the formation of ammonium. The genome of strain 9FUS^T^ contains the *nifVHDKBENBA* operon (AB6M95_00645-00710), encoding the molybdenum–iron nitrogenase and auxiliary genes and orthologous to the one previously described for *P. methanolicus* strain 5S69^T^ [21]. However, the genome of strain 9FUS^T^ also contains the *anfHDKGOR* gene cluster (AB6M95_01005-01040), coding an alternative iron–iron nitrogenase. This enzyme and its structural genes have originally been described for *Azotobacter vinelandii* [72]. The iron–iron nitrogenase of *Rhodobacter capsulatus* was recently shown to reduce not only N_2_ to NH_4_ but also CO_2_ to methane and formate; it was less selective to dinitrogen than the Mo- and V-nitrogenases [73].

**Figure 7:**
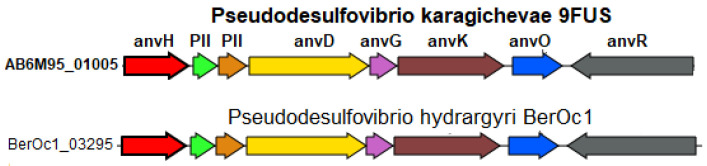
Organization of gene clusters presumably encoding the alternative nitrogen fixation enzyme in the genome of strain 9FUS^T^. Abbreviations: *anvH*, nitrogenase (iron–iron) reductase and maturation protein; PII, Nitrogen regulatory protein P-II, nitrogen-fixation associated; *anvD*, Nitrogenase (iron–iron) alpha chain; *anvG*, nitrogenase (iron–iron) delta chain; *anvK*, nitrogenase (iron–iron) beta chain; *anvO*, unknown; *anvR*, nitrogenase (iron–iron) transcriptional regulator.

Among members of the genus *Pseudodesulfovibrio*, a homologous cluster of alternative nitrogenase genes was found only in the genome of *P. hydrargyri* BerOc1^T^ (Figure 7). Among the three known nitrogenase types, the Fe-only nitrogenase is considered the simplest since its function depends on fewer gene products compared to the orthologous but more complex Mo- and V-nitrogenases. Its investigation holds, therefore, a biotechnological promise [74].

Duplication of substrate utilization pathways in strain 9FUS^T^ probably provides for its metabolic lability, improving its adaptation to its environment.

### 3.6. Ecological Implications

The genus *Pseudodesulfovibrio* was described in 2016 [24], and it currently comprises 13 validly published species and 5 species not validly published under the International Code of Nomenclature of Bacteria (ICNP) [https://lpsn.dsmz.de/genus/pseudodesulfovibrio (accessed on 15 October 2024)]. This genus belongs to the family *Desulfovibrionaceae*, order *Desulfovibrionales*, class *Deltaproteobacteria* of the phylum *Pseudomonadota* [24,38]. Members of this genus were isolated from deep-sea hydrothermal areas, brackish lake sediments, terrestrial mud volcanoes, oil refinery water, and production water of petroleum reservoirs [15,21].

According to the BLAST analysis of the GenBank database on a tree including the 16S rRNA gene of the strain 9FUS^T^ and 99 other closely related sequences, the strain 9FUS^T^ forms a single cluster with several bacteria of the genera *Pseudodesulfovibrio* and *Desulfovibrio* (Figure S7). The sequences of *Pseudodesulfovibrio* sp. strain 09 and 9ES were isolated from the same habitat as strain 9FUS^T^, and *Desulfovibrio* sp. HQM3 isolated from mudflat turned out to be the closest. The more distant sequences of this cluster belonged to *Pseudodesulfovibrio* sp. 09S isolated from the same habitat, as well as *Desulfovibrio* sp. Z1 isolated from seawater and *Desulfovibrio caledoniensis* SEBR 7250 isolated from oil field brines. Thus, SRBs that are taxonomically close to the strain under study (9FUS^T^) are found not only in oil reservoirs but also in other habitats.

Sulfate-reducing strains of the genus *Pseudodesulfovibrio* (9FUS^T^, 9ES, 09, DNS2, and 09S) and *Oleidesulfovibrio alaskensis* DNS2 were isolated at the temperature, pH, and salinity of their environment (Karazhanbas oil field) and can be considered as an indigenous microbiota to the oil reservoir. They are able to use lower alcohols (methanol, ethanol) and some sugars and grow on H_2_/CO_2_ in the presence of acetate. The latter can be formed from H_2_/CO_2_ by homoacetogenic bacteria *Acetobacterium carbinolicum* 9FOS, isolated from the same formation. When sulfates enter the reservoir with injected water, sulfate reducers can receive energy by their reduction to sulfide. However, at the time of the study, sulfate was not registered in reservoir water, which led to a low representation of sulfate reducers in the microbial community [15] and the predominance of methanogens as the final destructors of the organic matter of oil. Under these conditions, sulfate reducers probably grow as chemoorganoheterotrophs, fermenting carbonaceous and proteinaceous substrates, including necromass (i.e., dead biomass), and contribute to carbon turnover. Genomic analysis of strain 9FUS^T^ confirms that amino acid metabolism and carbohydrate metabolism are the major catabolic pathways in *Pseudodesulfovibrio* 9FUS^T^. Apart from sulfate reducers, acetogenic and methanogenic prokaryotes occurring in the reservoir may also contribute to the corrosion of the steel equipment. The corrosive activity of these prokaryotic groups has been demonstrated previously [12,13,14].

Strain 9FUS^T^ was not found to possess the gene encoding alkylsuccinate synthase (Ass) responsible for the anaerobic degradation of alkanes and the genes encoding anaerobic degradation of the aromatic compounds present in petroleum (benzoate, phenol, catechol, and meta- and ortho-cleavage). However, in the 9FUS^T^ genome were present many genes encoding the pathways related to anaerobic respiration of oxidized sulfur compounds, important for the development of microbial corrosion. The sulfide formed by sulfate-reducing bacteria can chemically interact with metals, causing extensive steel corrosion.

There is extensive literature on the negative effects of bacteria on steel equipment in the oil and gas industry [2,11,12,13,75,76].

The production of an extracellular polymer matrix and the formation of biofilms, the presence of flagella and pili can contribute to the growth of the studied strain and other SRB on the surface of metals and to pitting corrosion of steel. The presence of pili in strain 9FUS^T^ is confirmed by the annotation of the *pilA* and *cpaABCEF* genes (AB6M95_10525-10550) encoding the formation of pili.

It was noted that the concentration of organic substrates affects the rate of corrosion [77,78,79]. In media with a high content of an organic substrate, sulfate-reducing bacteria preferentially use it and increase the biomass, decreasing the corrosion rate and pitting depth. These results indicate that the corrosion rate has no direct correlation with the number of SRBs [79]. In the absence of organic compounds, these bacteria are capable of obtaining energy using elemental iron as the electron donor for sulfate reduction [80].

Thakur and co-workers analyzed 63 genomes of bacteria of the genus *Desulfovibrio* and identified the genes and proteins participating in the biocorrosion process by sulfate-reducing bacteria [81]. The genes determining the metal ion binding and sulfur metabolism, *hysB* and *hydA*, and *sat* and *dsr*, respectively, were found in all 63 genomes. These genes were also present in the genome of strain 9FUS^T^ and other bacteria of the genus *Pseudodesulfovibrio*, which includes the species formerly assigned to *Desulfovibrio*.

The most important property of the strain 9FUS^T^, which determines its potential participation in biocorrosion, is its ability to grow on molecular hydrogen. In the 9FUS^T^ genome were present genes *echABCDEF* (AB6M95_12990-13015) and *hynAB* (AB6M95_09170-09175; 045-85-04590) encoding hydrogenases, characteristic of other bacteria of the genus *Pseudodesulfovibrio*. However, the *cooMKLXUH* operon (AB6M95_00505-00550) is annotated in the genome of strain 9FUS^T^, presumably encoding a rare for this genus membrane Coo-carbon monoxide-induced [Ni-Fe] hydrogenase. A set of hydrogenases is assumed based on the analysis of the genome of strain 9FUS^T^, possibly participating in removing hydrogen from the metal through electron transfer to sulfate, which contributes to electrochemical corrosion and pitting formation.

2026 marks the 100th anniversary of the appearance of papers by E.S. Bastin [82] and T.L. Ginzburg-Karagicheva [83] dealing with sulfate-reducing bacteria in oil fields. These authors are considered the founders of petroleum microbiology as a science. The bacterium *Desulfovibrio bastinii* (at present *Maridesulfovibrio bastinii*) is named after E.S. Bastin [84]. To pay tribute to Tatiana L. Ginzburg-Karagicheva’s contribution to petroleum microbiology, we named a new bacterium in her honor–*Pseudodesulfovibrio karagichevae* sp. nov.

## 4. Conclusions

Metagenomic studies have revealed the distribution of *Pseudodesulfovibrio* members in petroleum reservoirs [21,37,43,56], including the Karazhanbas oil field located in Kazakhstan [15]. In the present work, five sulfate-reducing strains identified as members of the genera *Pseudodesulfovibrio* and *Oleidesulfovibrio* were isolated from the Karazhanbas oil field. Four strains with high 16S rRNA gene similarity between each other probably belonged to a new species of the genus *Pseudodesulfovibrio.* An in-depth study of strain 9FUS^T^, which was chosen as the type strain, revealed the set of phenotypic features distinguishing this strain from its closest relatives, ‘*P. methanolicus*’, *P. hydrargyri*, *P. mercurii*, and ‘*P. thermohalotolerans*’. These phenotypic features include the ability to grow without NaCl and to use ribose, methanol, ethanol, malate, and succinate, but not lactose, as electron donors. Values of overall genomic relatedness indices (OGRI) of strain 9FUS^T^, ANI, and dDDH, with genomes of *Pseudodesulfovibrio* species, were below 91.6% and 45.0%, respectively. Based on the distinct phylogenetic position of the strain 9FUS^T^ on concatenated proteins and 16S rRNA gene trees, this strain was assigned to a new species of the genus *Pseudodesulfovibrio*, for which the name *Pseudodesulfovibrio karagichevae* sp. nov. is proposed (=VKM B-3654^T^ = KCTC 25498^T^). The description of the species *Pseudodesulfovibrio karagichevae* sp. nov. is given in the protologue (Table 4).

## Figures and Tables

**Table 1:** Taxonomic affiliation of sulfidogenic bacterial strains isolated from the Karazhanbas oil field.

Strain	GenBank Number of 16S rRNA Gene Sequence	Closest Type Strain According to 16S rRNA Gene, acc. no.	16S rRNA Gene Similarity, %	Substrate/Acceptor
9FUS^T^	PQ453521	*Pseudodesulfovibrio mercurii* ND132, CP003220	98.6	Fumarate/S^0^
9ES	PQ453522	*P. mercurii* ND132, HQ693571	98.4	Ethanol/SO_4_^2−^
09	PQ453523	*P. mercurii* ND132, CP003220	98.7	H_2_ + CO_2_/SO_4_^2−^
09S	PQ474646	*P. mercurii* ND132, CP003220	98.5	Fumarate/S^0^
DNS2	PQ453525	*Oleidesulfovibrio alaskensis* Al1, NR_029338	100	H_2_ + CO_2_ + acetate/SO_4_^2−^
9FOS	PQ453524	*Acetobacterium carbinolicum* DSM 2925, NR_026325.1	99.1	Formate/S^0^

**Table 2:** The differentiating characteristics of strain 9FUS^T^ and some *Pseudodesulfovibrio* species. Strains: 1, 9FUS^T^ (this study); 2, *P. methanolicus* 5S69^T^ [21]; 3, *P. hydrargyri* BerOc1^T^ [42]; 4, *P. mercurii* ND132^T^ [41]; 5, ‘*P. thermohalotolerans*’ MCM B-1480^T^ [43].

Characteristic	Type Strains				
	9FUS^T^	5S69^T^	BerOc1^T^	ND132^T^	MCM B-1480^T^
	1	2	3	4	5
NaCl % (*w*/*v*) range (optimum)	0–5 (0–2)	0.2–6 (2–4)	0.2–4.0 (1.5)	0–3.0 (2.0)	1.0–6.0 (3.0)
Temperature (°C) range (optimum)	15–37 (23–28)	15–37 (23–28)	25–35 (30)	20–37 (32)	20–60 (37)
pH range (optimum)	4.1–8.6 (6.5)	4.6–8.6 (6.5)	(6.0–7.4)	6.8–8.2 (7.8)	6.0–8.0 (7.0)
Electron donor with sulfate:					
H_2_/CO_2_	+ *	+ *	+	+ *	ND
Formate	+	+	−	+ *	+
Succinate	+	+	−	−	+
Fumarate	+	+	+	+	+
Citrate	−	W	−	ND	−
Malate	+	+	−	−	ND
Benzoate	−	−	−	ND	ND
Methanol	+	+	−	−	ND
Ethanol	+	+	W	−	ND
Glycerol	+	+	−	ND	ND
Glucose	−	−	−	−	+
Sucrose	−	−	−	ND	+
Fructose	+	+	−	ND	+
Lactose	−	−	−	ND	+
Galactose	W	W	−	ND	+
Ribose	+	−	−	ND	ND
Electron acceptor:					
Elemental sulfur	+	+	−	ND	−
Nitrate	−	−	−	−	+
Fermentation of:					
Lactate	−	−	−	−	+
Fumarate	−	+	−	+	+
Genome size (Mb)	4.20	4.16	4.1	3.86	3.9
Genomic G + C content (%)	64.0	63.0	64.0	65.2	60.5
Major cellular fatty acids	*i*-C_17:1_ ω11, C_15:0_, *i*-C_15:0_, C_16:0_	*i*-C_15:0_, *ai*-C_15:0,_ C_16:0_	C_18:0_, *ai*-C_15:0_, C_16:0_, C_18:1_ ω7	*i*-C_15:0_, *ai*-C_15:0_, *i*-C_17:0_	*ai*-C_15:0_, *i*-C_15:0_, C_16:0_, *ai*-C_17:0_
Isolation source	Hydrocarbon reservoir	Hydrocarbon reservoir	Brackish lagoon sediments	Brackish bottom sediments	Hydrocarbon reservoir

All strains were obligately anaerobic, Gram-stain-negative, non-spore-forming, and motile. All strains were able to ferment pyruvate and utilize lactate and pyruvate, but not acetate, as substrates for sulfate reduction and use sulfate, sulfite, and thiosulfate as electron acceptors. Designations: ‘+’, positive reaction; (W), weakly positive reaction; ‘−’, negative reaction; ND, not determined. *, Growth was observed in the presence of acetate.

**Table 3:** The genome-relatedness indices (%) between strain 9FUS^T^ and type strains of the genus *Pseudodesulfovibrio*.

Type Strain	Genome	Ref.	Genome Size, Mb	G + C Content, mol.%	Strain 9FUS^T^		
					16S rRNA	dDDH	ANI
Strain 9FUS^T^	GCF_041414475.1		4.20	64.0	100.0	100.0	100.0
‘*P. methanolicus*’ 5S69^T^	GCF_037094465.1	[21]	4.16	63.1	98.9	45.0	91.6
*P. hydrargyri* BerOc1^T^	GCF_001874525.1	[42]	4.08	64.0	98.5	41.2	90.6
*P. mercurii* ND132^T^	GCF_000189295.2	[41,46]	3.86	65.2	98.2	35.3	88.6
‘*P. thermohalotolerans*’ MCM B-1480^T^	GCF_021353295.2	[43]	3.89	60.4	98.3	27.9	84.5
*P. indicus* J2^T^	GCF_004365595.1	[47]	3.96	63.5	98.0	26.8	84.0
*P. portus* JCM 14722^T^	GCF_026000375.1	[48,49]	3.40	61.2	96.5	22.5	80.1
‘*P. pelocollis*’ SB368^T^	GCF_030282845.1	[50]	3.43	63.7	95.4	21.0	78.3
*P. aespoeensis* Aspo-2^T^	GCF_000176915.2	[51,52]	3.63	62.6	95.9	20.9	78.2
‘*P. cashew*’ SRB007^T^	GCF_009762795.1	[53]	3.91	59.9	95.4	21.0	78.2
*P. alkaliphilus* F-1^T^	GCF_009729555.1	[54]	3.23	61.9	95.3	20.2	77.1
*P. sediminis* SF6^T^	GCF_020886695.1	[55]	3.76	54.0	96.7	19.7	76.0
*P. tunisiensis* RB22^T^	GCF_022809775.1	[56,57]	3.61	59.4	93.5	18.7	75.1
*P. senegalensis* DSM 101509^T^	GCF_008830225.1	[58,59]	3.37	58.1	92.8	18.5	74.0
*P. nedwellii* SYK^T^	GCF_027923765.1	[60,61]	3.76	49.4	96.7	18.5	73.9
*P. piezophilus* C1TLV30^T^	GCF_000341895.1	[62,63]	3.65	50.0	95.7	19.2	72.5

**Table 4:** Protologue description of *Pseudodesulfovibrio karagichevae* sp. nov.

Parameter	*Pseudodesulfovibrio karagichevae* sp. nov.
Genus name	*Pseudodesulfovibrio*
Species name	*Pseudodesulfovibrio karagichevae*
Species status	sp. nov.
Species etymology	ka.ra’gi.che.vae. N.L. gen. n. *karagichevae*, named in honor of the Russian microbiologist Tatiana L. Ginzburg-Karagicheva, who studied sulfate-reducing bacteria from oilfields in 1926 and is one of the founders of petroleum microbiology
Designation of the Type Strain	9FUS^T^
Strain Collection Numbers	VKM B-3654^T^ = KCTC 25498^T^
Genome accession number	GCF_041414475.1
Genome status	Contig
Genome size	4.20 Mb
G + C mol%	64.0
16S rRNA gene accession nr.	PQ453521
Description of the new taxon and diagnostic traits	The cells are strictly anaerobic, chemoorganotrophic, Gram-stain-negative, non-spore-forming, motile, slightly curved rods. Mesophilic, with a growth range of 15–37 °C (optimum, 23–28 °C). Growth is observed in the presence of 0–5% (*w*/*v*) NaCl (optimum, 0–2% NaCl), at pH 4.1–8.6 (optimum, pH 6.5). Lactate, pyruvate, formate, malate, fumarate, succinate, methanol, ethanol, glycerol, fructose, ribose, yeast extract, and H_2_/CO_2_ (in the presence of acetate) are used as carbon and energy sources for sulfate reduction; weak growth occurs on glutamate, propanol, galactose, and mannose, but acetate, propionate, butyrate, citrate, glycine, L-serine, ornithine, glucose, lactose, sucrose, and benzoate are not used. No autotrophic growth. Lactate is oxidized to acetate and CO_2_. Fermentative growth occurs with pyruvate, but lactate is not fermented in the absence of terminal electron acceptors. Reduces sulfate, sulfite, thiosulfate, and elemental sulfur to sulfide in the presence of lactate but does not use nitrate. The predominant cellular fatty acids are *iso*-C_17:1_ ω11, C_15:0_, *iso*-C_15:0_, and C_16:0_. The major polar lipids are phosphatidylethanolamine, diphosphatidylglycerol, and phosphatidylglycerol. The major respiratory quinone is menaquinone MK-6(H_2_). The genome size of the type strain is 4.20 Mb with a genomic G + C content of 64.0 mol%. The type strain, 9FUS^T^ (VKM B-3654^T^ = KCTC 25498^T^), was isolated from the Karazhanbas oil field in Mangystau Province, The Republic of Kazakhstan. The GenBank/EMBL/DDBJ accession number for the 16S rRNA gene sequence is PQ453521, and the genomic assembly accession number is GCF_041414475.1.
Country and region of origin	The Republic of Kazakhstan, Mangystau Province
Date of isolation	2019
Source of isolation	Production water from the Karazhanbas oil field
Sampling date	June 2019
Latitude, Longitude	43°22′33.3″ N, 52°59′27.1″ E
Depth (meters below sea level)	350
Number of strains in study	4
Information related to the Nagoya Protocol	Not applicable

## Data Availability

The whole-genome shotgun project of strain 9FUS^T^ has been deposited at DDBJ/EMBL/GenBank under the accession GCF_041414475.1, and it is the first version described in this paper.

## Supplementary Materials

The following supporting information can be downloaded at: https://www.mdpi.com/article/10.3390/microorganisms12122552/s1, Figure S1: Growth profiles of strain 9FUS^T^ in the lactate-sulfate medium at various temperatures (a), NaCl concentrations (g·L^−1^) (b), and pH (c) after 14 days of incubation; Figure S2: Thin layer chromatograms of polar lipids from the strains 9FUS^T^ and ‘*Pseudodesulfovibrio methanolicus*’ 5S69^T^ (b, [21]). The components were visualized by staining with 5% sulfuric acid in ethanol and heating at 180 °C for 15 min. Abbreviations: PE, phosphatidylethanolamines; DPG, diphosphatidylglycerols; PG, phosphatidylglycerols; GL, glycolipids; PL, phospholipids; APL, aminophosholipids; LPE, lysophosphatidylethanolamines; GPL, glycophospholipids; Figure S3: Identification of polar lipids from the strains 9FUS^T^ (A) and 5S69^T^ (B, [21]). The components were visualized by molybdenum blue (a), α-naphthol (b), ninhydrin (c), and Dragendorff’ reagent (d). Abbreviations as in Figure S2; Figure S4: KEGG-map of the “Pentose phosphate pathway” based on the genome analysis of the strain 9FUS^T^. The presumptive pathway of ribose assumption is highlighted in red. The enzymes annotated in the genome are highlighted in green as in Figures S5 and S6; Figure S5: KEGG-map of the “Fructose and mannose metabolism” pathway based on the genome analysis of strain 9FUS^T^. The presumptive pathway of fructose assumption via fructokinase is highlighted in blue, and via 1-phosphofructokinase is highlighted in red; Figure S6: KEGG-map of the “Glycerolipid metabolism” pathway based on the genome analysis of strain 9FUS^T^. The presumptive pathway of glycerol assumption is highlighted in red; Figure S7: Phylogenetic tree of 16S rRNA gene sequences of the 99 bacterial strains closest to the strain 9FUS^T^ according to a BLAST analysis based on GenBank. The cluster of the closest strains is marked with a red asterisk; Table S1: Physicochemical characteristics of reservoir water* from the production well 6069 of the Karazhanbas oil field; Table S2: Cellular fatty acid composition of strain 9FUS^T^ and the type strain of ‘*Pseudodesulfovibrio methanolicus*’ 5S69^T^.
