# Supplementary material for: Sulfate-Reducing Bacteria Isolated from an Oil Field in Kazakhstan and a Description of Pseudodesulfovibrio karagichevae sp. nov
Source: Microorganisms. 2024 Dec 11;12(12):2552. doi: 10.3390/microorganisms12122552 (PMC11678503; doi:10.3390/microorganisms12122552)
Supplement: Supplementary file 1 [file microorganisms-12-02552-s001.zip › microorganisms-3319699-supplementary.pdf]

microorganisms

MDPI

# Supplementary Materials

# Sulfate-Reducing Bacteria Isolated from an Oil Field in Kazakhstan and a Description of *Pseudodesulfovibrio karagichevae* sp. nov.

**This file includes:**

Figures S1 to S6

Tables S1 and S2

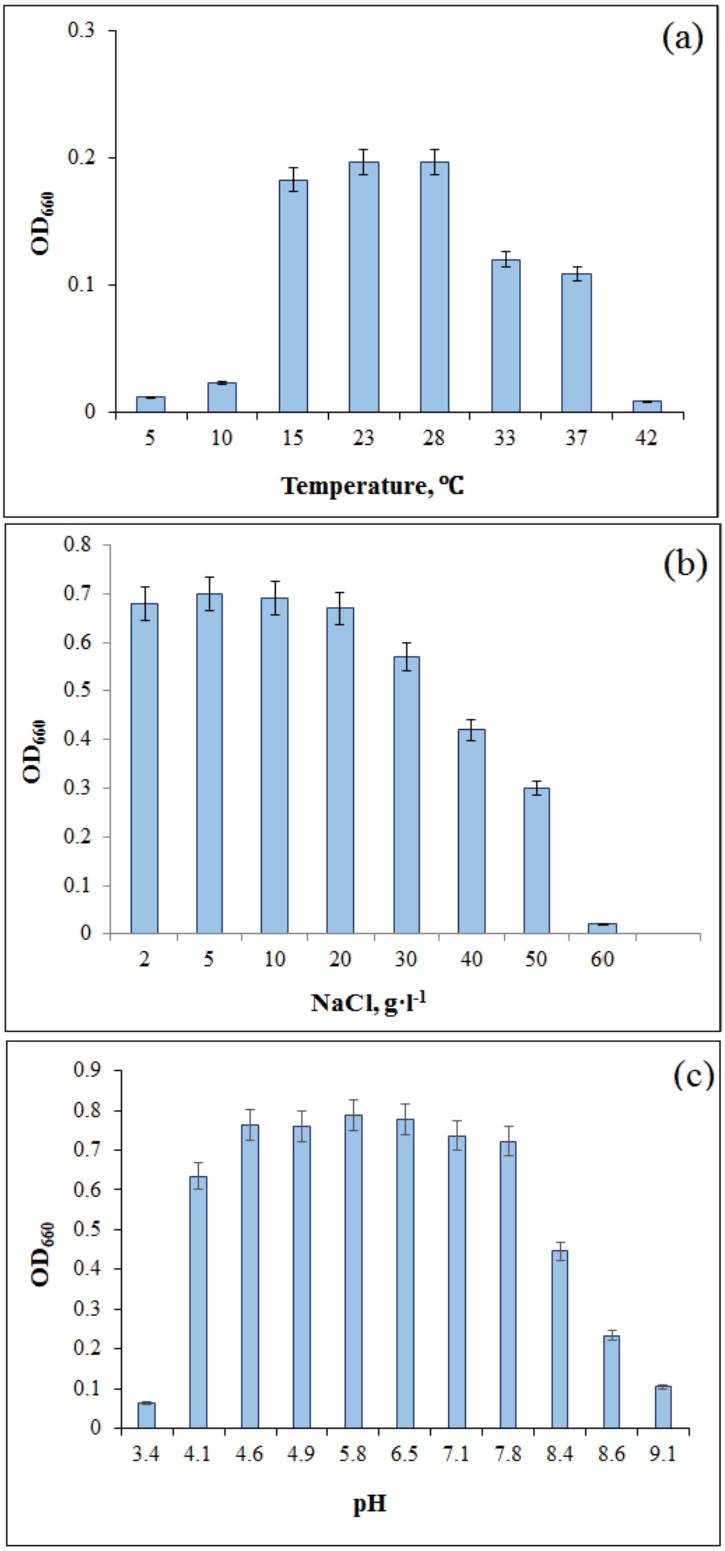


**Figure S1.** Growth profiles of strain 9FUS$^T$ in the lactate-sulfate medium at various temperatures (a), NaCl concentrations ($g \cdot L^{-1}$) (b), and pH (c) after 14 days incubation.

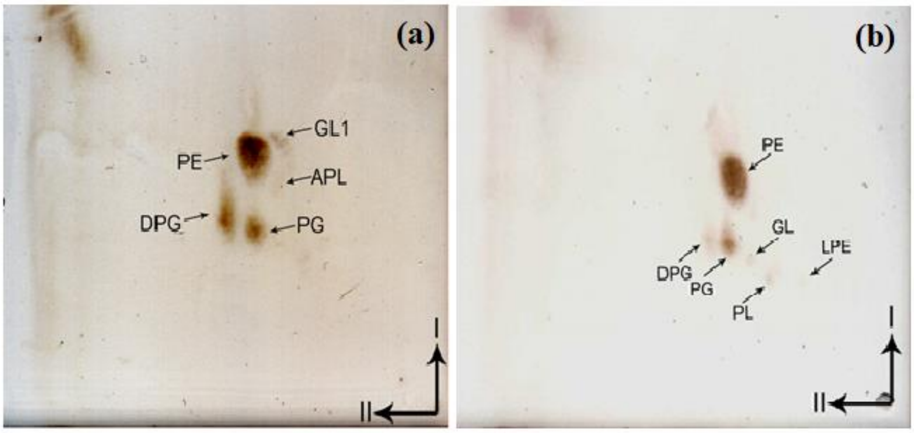


**Figure S2.** Thin layer chromatograms of polar lipids from the strains 9FUS$^{T}$ and '*Pseudodesulfovibrio methanolicus'* 5S69$^{T}$ (b, [21]). The components were visualized by staining with 5% sulfuric acid in ethanol and heating at 180 °C for 15 min. Abbreviations: PE, phosphatidylethanolamines; DPG, diphosphatidylglycerols; PG, phosphatidylglycerols; GL, glycolipids; PL, phospholipids; APL, aminophosholipids; LPE, lysophosphatidylethanolamines; GPL, glycophospholipids.

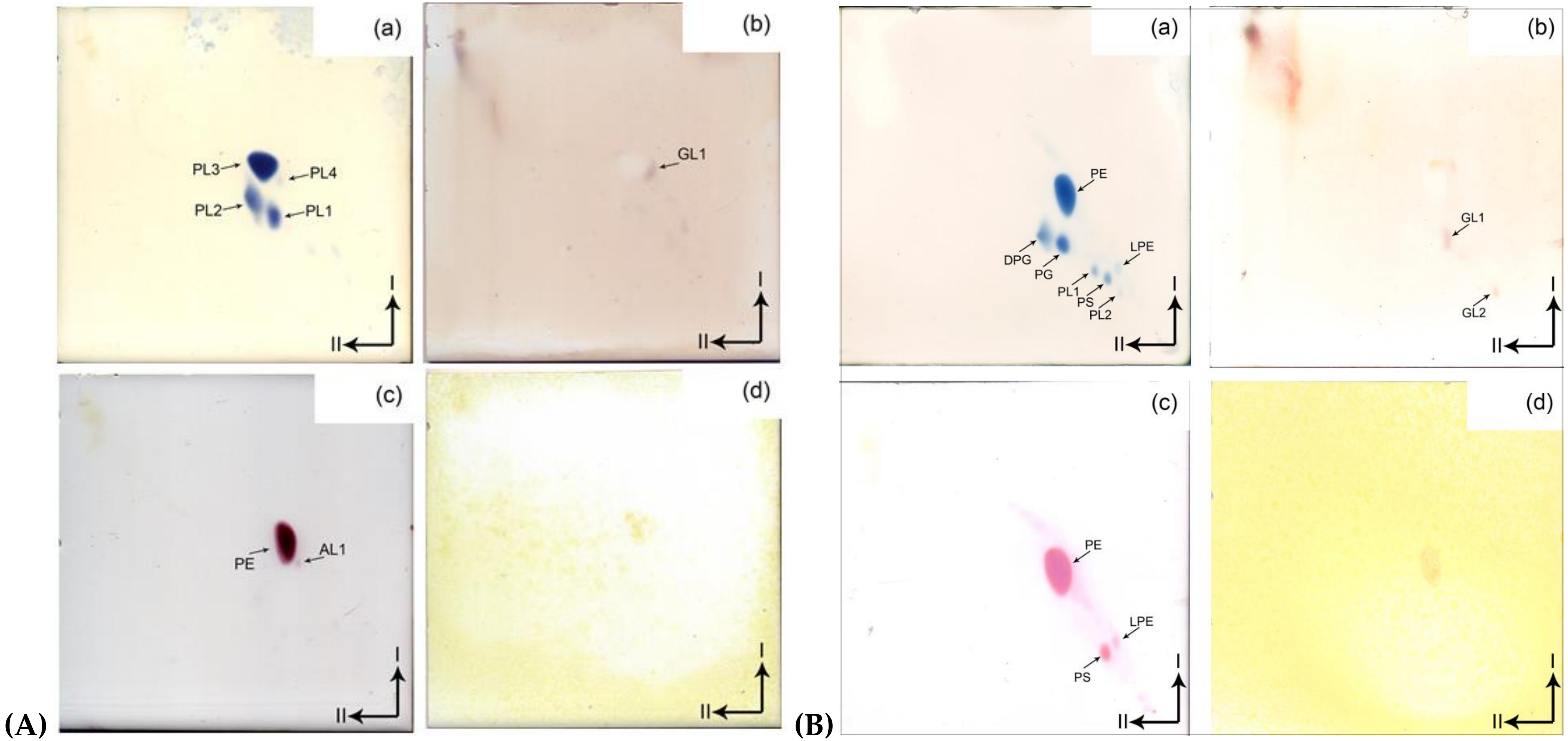


**Figure S3.** Identification of polar lipids from the strains 9FUS$^T$ (A) and 5S69$^T$ (B, [21]). The components were visualized by molybdenum blue (a), $\alpha$-naphthol (b), ninhydrin (c), and Dragendorff' reagent (d). Abbreviations as in Figure S2.

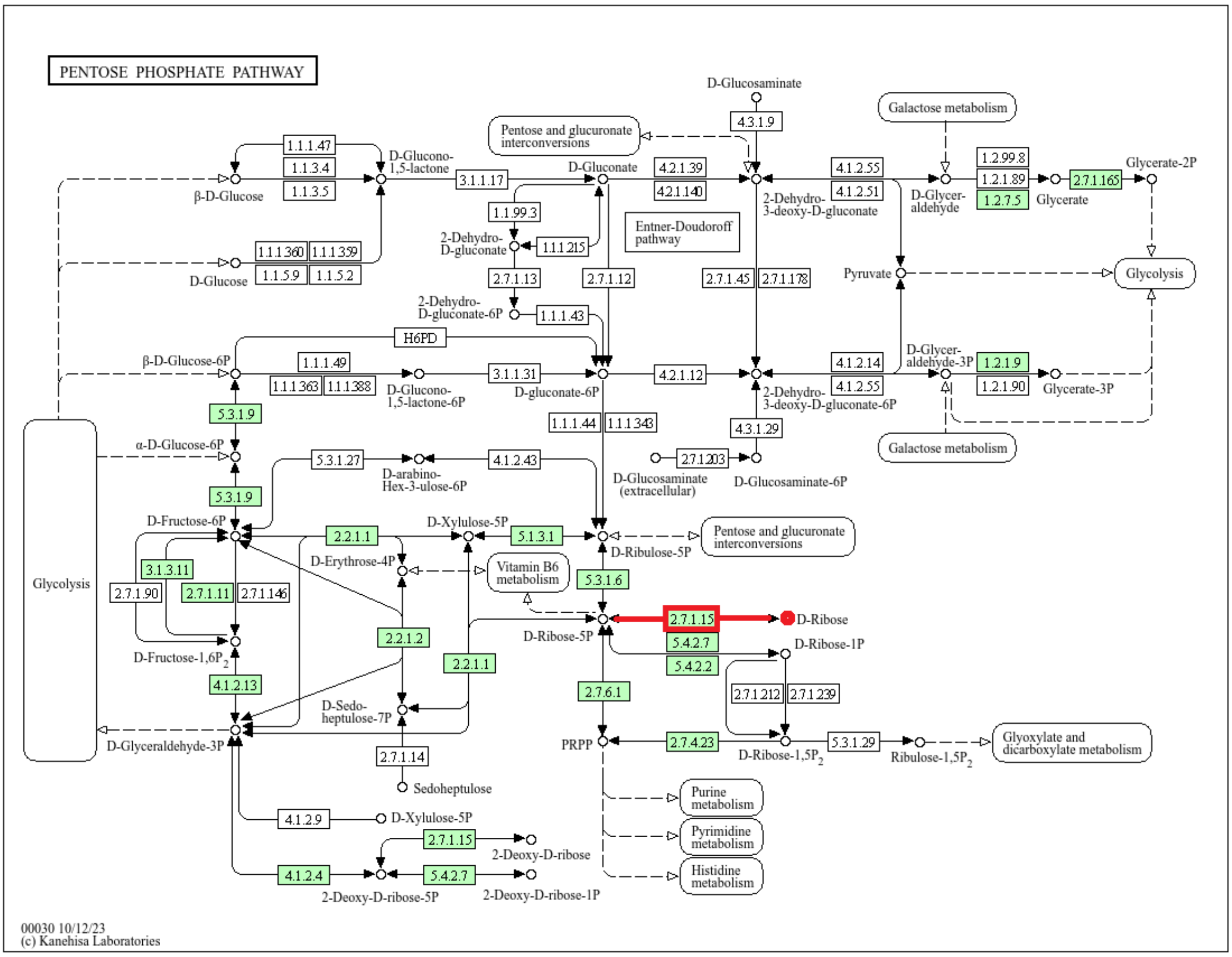


**Figure S4.** KEGG-map of the "Pentose phosphate pathway" based on the genome analysis of the strain 9FUS$^{T}$. The presumptive pathway of ribose assumption is highlighted in red. The enzymes annotated in the genome are highlighted in green as in Figures S5–S6.

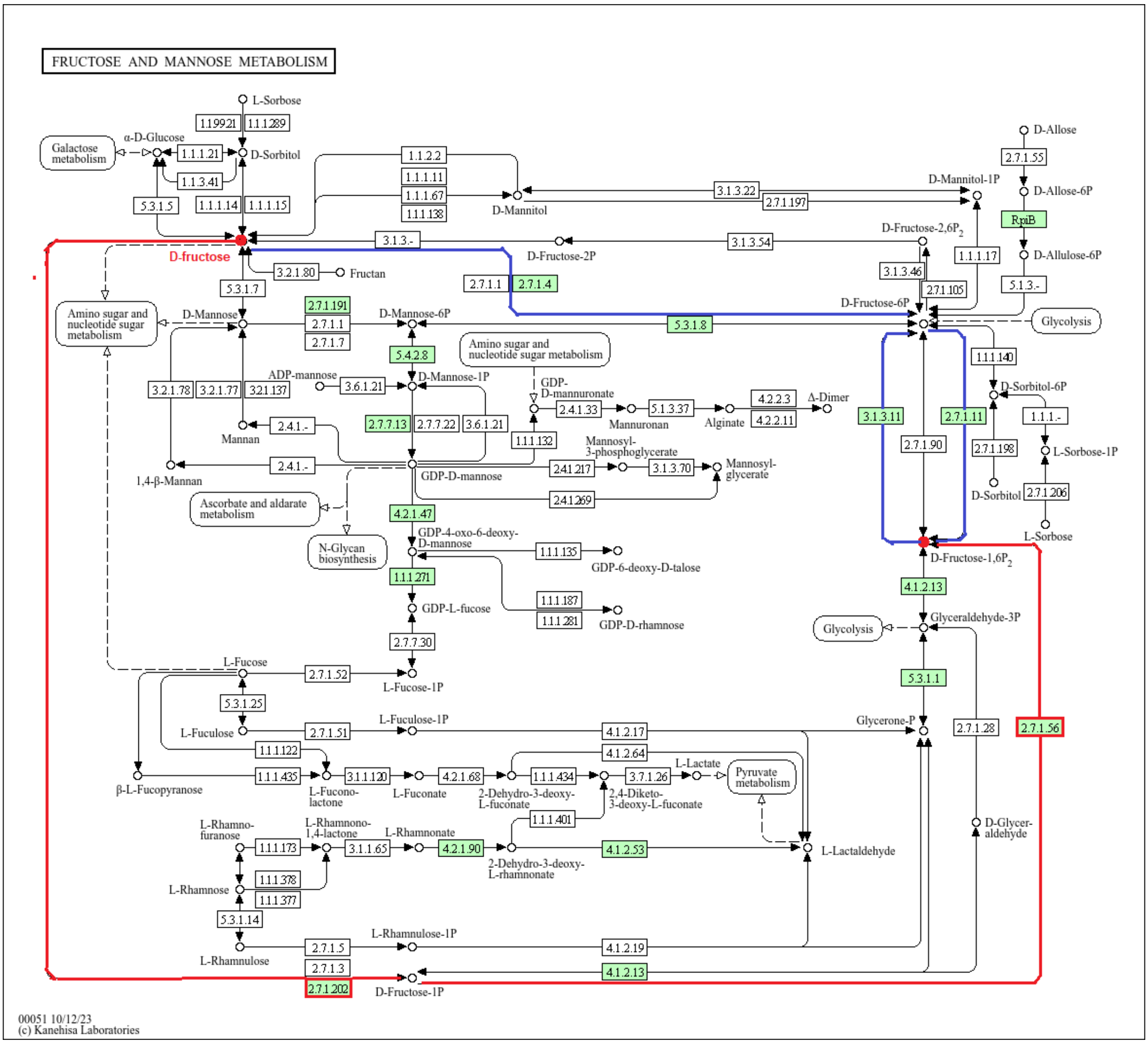


**Figure S5.** KEGG-map of the "Fructose and mannose metabolism" pathway based on the genome analysis of strain 9FUS[T]. The presumptive pathway of fructose assumption via fructokinase is highlighted in blue and via 1-phosphofructokinase is highlighted in red.

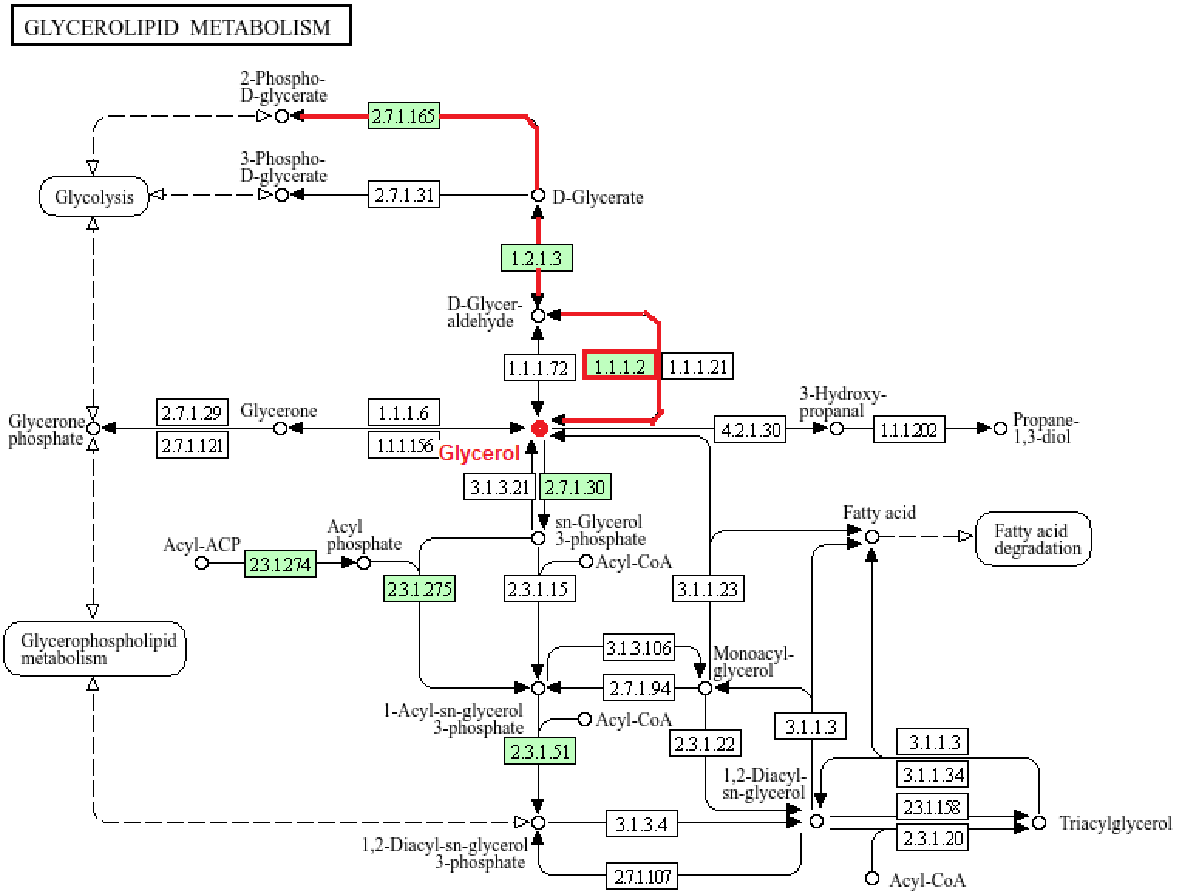


**Figure S6.** KEGG-map of the "Glycerolipid metabolism" pathway based on the genome analysis of strain 9FUS$^T$. The presumptive pathway of glycerol assumption is highlighted in red.

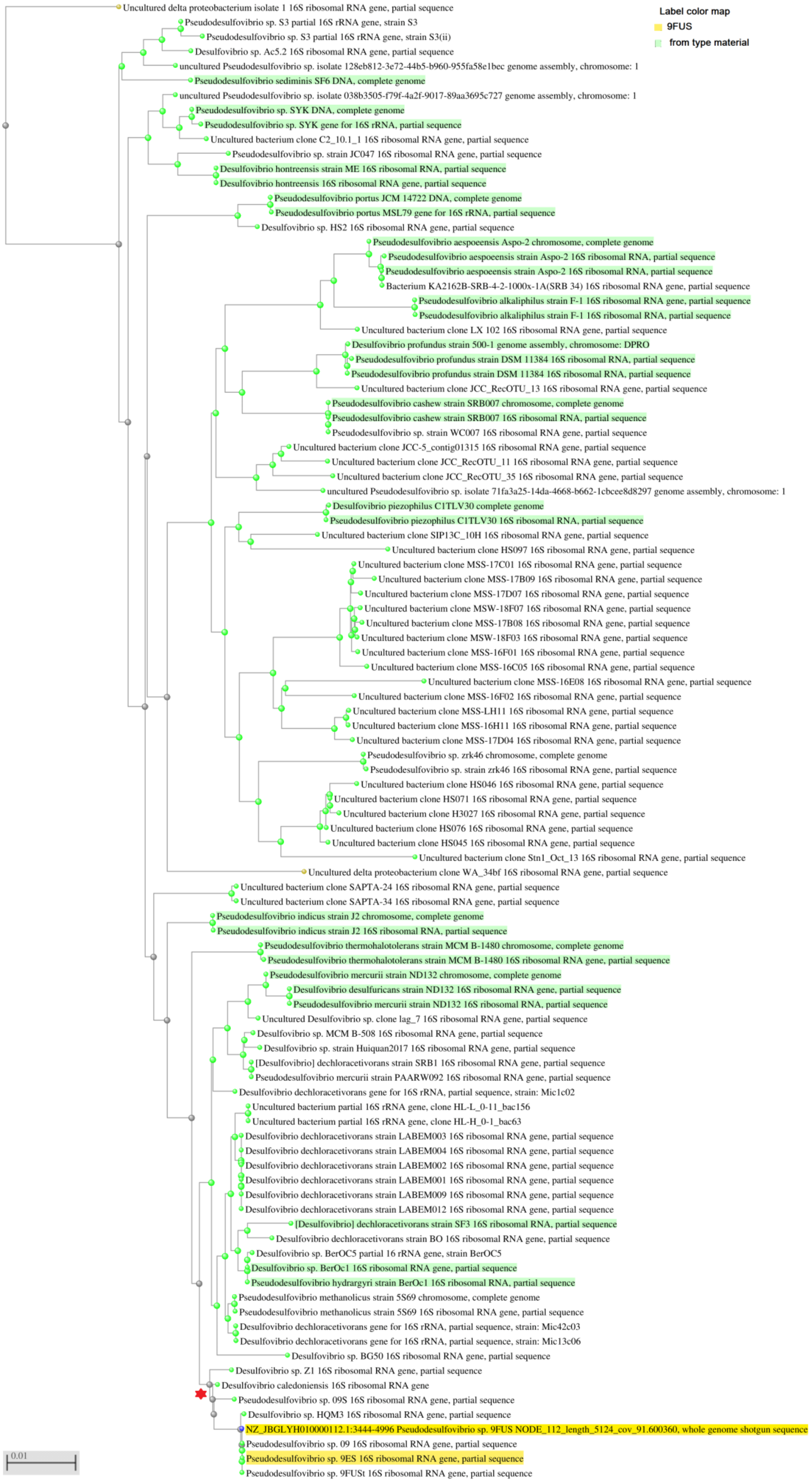


**Figure S7.** Phylogenetic tree of 16S rRNA gene sequences of the 99 bacterial strains closest to the strain 9FUS$^{T}$ according to a BLAST analysis based on GenBank. The cluster of the closest strains is marked with a red asterisk.

**Table S1.** Physicochemical characteristics of reservoir water* from production well 6069 of the Karazhanbas oil field.

| Total salinity, $mg{\cdot}L^{-1}$ | Content, $mg{\cdot}L^{-1}$ | | | | | | | |
|---|---|---|---|---|---|---|---|---|
| | $Na^{+}+K^{+}$ | $Ca^{2+}$ | $Mg^{2+}$ | $Cl^{-}$ | $SO_4^{2-}$ | $CO_3^{2-}$ | $HCO_3^{-}$ | $H_2S$ |
| 32959.2 | 10221.2 | 1202.4 | 851.2 | 19915.8 | 0 | 0 | 768.6 | 62.0 |

**Table S2.** Cellular fatty acid composition of strain 9FUS$^T$ and the type strain of '*Pseudodesulfovibrio methanolicus*' 5S69$^T$.

| **Fatty acid** | **Strain 9FUS$^T$** | **Strain 5S69$^T$** |
|---|---|---|
| iso-$C_{14:0}$ | – | 3.2 |
| $C_{14:0}$ | 1.8 | 2.8 |
| iso-$C_{15:0}$ | **9.0** | **20.4** |
| anteiso-$C_{15:0}$ | 0.4 | **19.3** |
| $C_{15:0}$ | **14.6** | 0.5 |
| iso-$C_{16:1}$ ω9 | – | 2.5 |
| iso-$C_{16:0}$ | – | 4.2 |
| $C_{16:1}$ ω9 | 0.8 | 0.5 |
| $C_{16:0}$ | 8.5 | **16.3** |
| iso-$C_{17:1}$ ω11 | **20.8** | – |
| $C_{17:1}$ ω10c | 3.2 | 2.9 |
| anteiso-$C_{17:1}$ ω9 | 4.2 | 1.2 |
| $C_{17:1}$ ω9c | 2.0 | 3.8 |
| $C_{17:1}$ ω9t | – | **6.6** |
| iso-$C_{17:0}$ | – | 3.5 |
| anteiso-$C_{17:0}$ | 3.5 | 2.6 |
| $C_{17:0}$ | 7.7 | – |
| $C_{18:1}$ ω11 | 2.3 | – |
| $C_{18:1}$ ω10 | – | 3.7 |
| $C_{18:1}$ ω9c | 6.0 | 1.8 |
| $C_{18:1}$ ω9t | – | 0.4 |
| $C_{18:1}$ ω8 | 1.1 | – |
| $C_{18:0}$ | 7.1 | 1.4 |
| $C_{19:1}$ ω10 | 2.9 | – |
| $C_{24:0}$ | 1.5 | – |
| Others | 2.6 | 2.4 |
| Total | 100.00 | 100.00 |

*The values are percentages (w/w) of total fatty acids. Dominant fatty acids are indicated in bold.
